# Abiotic ammonium formation in the presence of Ni-Fe metals and alloys and its implications for the Hadean nitrogen cycle

**DOI:** 10.1186/1467-4866-9-5

**Published:** 2008-05-19

**Authors:** Alexander Smirnov, Douglas Hausner, Richard Laffers, Daniel R Strongin, Martin AA Schoonen

**Affiliations:** 1 Department of Geosciences, Stony Brook University, Stony Brook, NY 11794, USA; 2 Department of Chemistry, Temple University, Philadelphia, PA 19122, USA

## Abstract

Experiments with dinitrogen-, nitrite-, nitrate-containing solutions were conducted without headspace in Ti reactors (200°C), borosilicate septum bottles (70°C) and HDPE tubes (22°C) in the presence of Fe and Ni metal, awaruite (Ni_80_Fe_20_) and tetrataenite (Ni_50_Fe_50_). In general, metals used in this investigation were more reactive than alloys toward all investigated nitrogen species. Nitrite and nitrate were converted to ammonium more rapidly than dinitrogen, and the reduction process had a strong temperature dependence. We concluded from our experimental observations that Hadean submarine hydrothermal systems could have supplied significant quantities of ammonium for reactions that are generally associated with prebiotic synthesis, especially in localized environments. Several natural meteorites (octahedrites) were found to contain up to 22 ppm N_tot_. While the oxidation state of N in the octahedrites was not determined, XPS analysis of metals and alloys used in the study shows that N is likely present as nitride (N^3-^). This observation may have implications toward the Hadean environment, since, terrestrial (e.g., oceanic) ammonium production may have been supplemented by reduced nitrogen delivered by metal-rich meteorites. This notion is based on the fact that nitrogen dissolves into metallic melts.

## Introduction

Ammonia (NH_3_) or ammonium (NH_4_^+^), henceforth NH_3_/NH_4_^+^, are necessary precursors for reactions associated with prebiotic syntheses, such as the Strecker synthesis. It has been experimentally shown that NH_3_/NH_4_^+ ^environments are more efficient in organic synthesis than those dominated by dinitrogen (henceforth N_2_) in both aqueous and gaseous environments [e.g., [1]] [2,3]. This notion is not unexpected, considering that, the strong triple bond (948 kJ.mol^-1^) of the N_2 _would presumably result in large reaction activation barriers (i.e., low conversion rates), even if the overall reaction is thermodynamically favored.

Several possible pathways to abiotic NH_3_/NH_4_^+ ^on early the Earth have been proposed: reduction of NO_2_^-^/NO_3_^- ^by Fe^++^/FeS in the ocean [e.g., [4]] [5,6]; atmospheric production from N_2 _and HCN [e.g., [7]] [8]; release from rocks and minerals [e.g., [9]]; photoreduction on mineral surfaces [e.g., [10]] [11,12]; and hydrothermal aqueous reduction from N_2 _in the presence of minerals under conditions typical of submarine hydrothermal systems [e.g., [13]] [14,15]. Each of the mechanisms relies on a different set of assumptions and none of the proposed mechanisms has, in our opinion, gained universal acceptance in the scientific community as the predominant source of abiotic NH_3_/NH_4_^+^.

**Figure 1:**
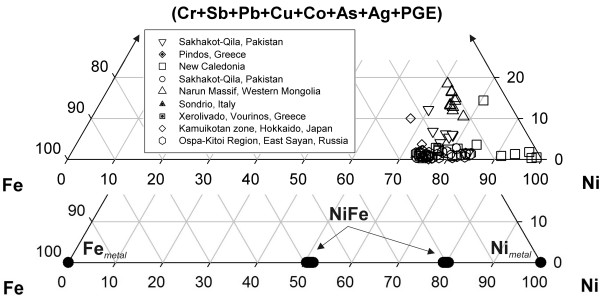
A ternary diagram of naturally occurring Ni-Fe-M (M = Cr, Sb, Pb, Cu, Co, As, Ag, PGE) alloys and their comparison to synthetic alloys used in this study. Analyses of natural samples were adapted from: [19] [22] [109-115]. Each data point represents an electron microprobe analysis expressed in weight percent.

In this scientific contribution we focus on the catalytic properties of Ni and Fe metals and their alloys which can form in submarine hydrothermal systems (SHS), especially those driven by exothermic hydration reactions (e.g., serpentinization) in an off-axis tectonic setting. Upon dissolution of Ni-containing rock-forming minerals (e.g., olivine, pyroxene, amphibole), released Ni and Fe can react to form metals and alloys under extreme reducing conditions imposed on the system by the serpentinization processes [16-18]. The conditions are commonly reducing enough to stabilize Ni-Fe alloys (e.g., awaruite – Ni_3_Fe), metallic nickel (Ni^0^) and even iron (Fe^0^). These minerals occur regularly, albeit in small quantities in both ancient and modern serpentinites [19-26]. A compilation of representative chemical analyses of metals and alloys found in serpentinites is presented in Fig. 1. The observations from natural systems have been corroborated by laboratory experiments [27,28].

An active global tectonic cycle is not required for the formation and operation of serpentinization-driven SHS and hence we assume that these environments were commonly present on the Hadean Earth. Moreover, the lack of oxygen and the possible presence of significant amounts of hydrogen gas in the Hadean atmosphere (and consequently in the ocean water) may have further enhanced the stability of base metals and their alloys [29,30].

The most abundant reactant for abiotic NH_3_/NH_4_+ formation in the Hadean was N_2 _dissolved in the seawater from the N_2_-rich atmosphere. NO_2_^- ^and NO_3_^- ^are also thought to have been available, although likely in low concentrations. These oxidized N species could have formed in high energy events such as lightning, corona discharge and/or impacts and subsequently rain out into the ocean [31-34].

In this contribution we report the results of an experimental study undertaken to evaluate the hypothesis that abiotic NH_4_^+ ^formation from dissolved N_2_, NO_2_^- ^and NO_3_^- ^in the presence of Ni_3_Fe, NiFe, Ni^0 ^and Fe^0 ^was an operative synthetic route at anaerobic conditions potentially present in the Hadean Ocean. Furthermore, we attempt to quantify global NH_4_^+ ^yields in the Hadean Ocean produced by investigated mechanisms.

## Methods and materials

### Reduction experiments

Three sets of experiments were conducted at three different temperatures: 200°C (runs 1–36), 70°C (runs 37–59) and 22°C (runs 60–83). The choice of reactors was based on experimental temperature: 15 mL passivated HIP^® ^Titanium 64 tube reactors (200°C); 20 mL I-Chem^® ^borosilicate vials with PTFE/Si septum caps (70°C) and 15 mL BD HDPE Falcon^® ^tubes (22°C). Reactors were kept at constant temperature in a heated water bath (20, 70°C) or an Isotemp^® ^oven (200°C). All experiments lasted 24 hours and were conducted in the absence of headspace (e.g., no gas phase). No additional pressure other than that of expanding liquid was imposed on the vessels (~400 psi/27 bars with Ni^0 ^to ~800 psi/55 bars with Fe^0 ^at 200°C).

All reacting solutions were prepared from freshly drawn UV/UF deionized water (henceforth DI) either by purging with a UHP gas of interest (e.g., Ar, N_2_, H_2_/N_2_) and/or by dissolving appropriate amounts of reagents. NH_4_^+ ^content of all unreacted reagent solutions (e.g., blanks) was below the detection limit of ion chromatography (<0.1 μmol.kg^-1^, reported by the manufacturer). DI purged with N_2 _gas under ambient conditions results in equilibrium N_2_(aq) concentration of 0.59 mmol.kg^-1^. The effect of O_2 _was, however, also determined (runs 39, 41, 45, 47, 50, 52, 56, 58, 62, 64, 68, 70, 74, 76, 80, 82) in experiments with DI equilibrated with present-day atmosphere (e.g., no N_2 _purging).

To ensure clean and fresh mineral surfaces (e.g., free of oxidation products and/or atmospheric sorbed gases), all metal/alloys were ultrasonically cleaned for 1 hour in 0.06 M HCl immediately preceding the experiments. Subsequently they were washed three times with the designated reacting solution and loaded into reactors in the form of slurry. This "wet loading" procedure eliminated sorption of gases from the atmosphere onto freshly cleaned metal surfaces which was especially important in blank experiments.

Background NH_4_^+ ^production (e.g., release from reactants, reaction vessels, etc.) was assessed in blank experiments with Ar-saturated solutions and no added N source (runs 1, 9, 17, 25, 37, 43, 49, 54, 60, 66, 72, 78). NO_3_^- ^and NO_2_^- ^solutions were prepared by dissolution of ACS reagent grade KNO_3 _and KNO_2_, respectively. The pH was not buffered and was allowed to change as a result of solution-metal/alloy interactions and was recorded before and after the experiment. After pH measurements, all samples were acidified with 0.2 M HCl to ensure the conversion of NH_3 _to NH_4_^+ ^and to prevent the formation of Fe precipitates. The samples were stored at 1°C and analyzed within 24 – 48 hours. The summary of all experimental conditions is presented in Tab. 1.

**Table 1:** Physical and chemical characteristics of alloys/metals used in study. Formula and the "*a*" parameter determined from EPMA analyses and from LeBail refinement of XRD data, respectively. The chemical composition of Ni-Fe alloys was confirmed by EPMA as: Ni_49.78(± 0.49)_Fe_50.22(± 0.49) _and Ni_79.14(± 0.43)_Fe_20.86(± 0.43)_.

*Metal/Alloy*	*Formula*	*Manufacturer*	*Surface Area [m^2^]*	*Space Group*	*Unit cell [Å]*	*Natural analog*
Iron	Fe	Alfa Aesar	0.6536 ± 0.0776	Im-3m	2.866(1)	Iron
Nickel	Ni	Alfa Aesar	0.5044 ± 0.0309	Fm-3m	3.523(1)	Nickel
Ni_50_Fe_50_	NiFe	Goodfellow	0.3685 ± 0.0094	Fm-3m	3.586(3)	Tetrataenite
Ni_81_Fe_19_	Ni_3.7_Fe	Goodfellow	0.1877 ± 0.0131	Fm-3m	3.547(6)	Awaruite

### Analysis of solids, their surfaces, and reaction products

**Figure 2:**
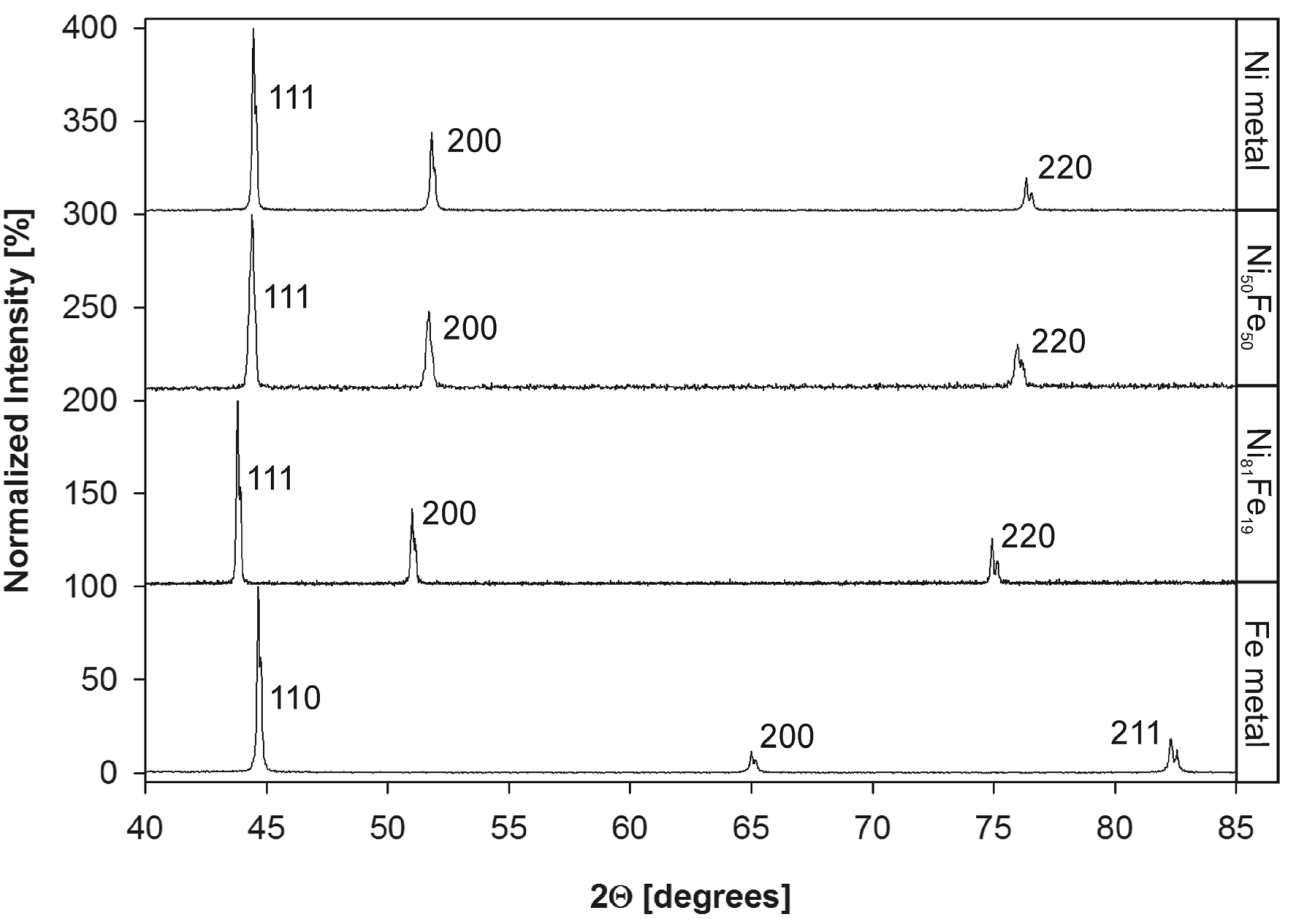
XRD patterns of metals/alloys used in this study. Note the similarity in patterns of Ni, Ni_50_Fe_50 _and Ni_81_Fe_19 _stemming from the same space group (Fm-3m). Fe^0 ^possesses Im-3m space group.

**Figure 3:**
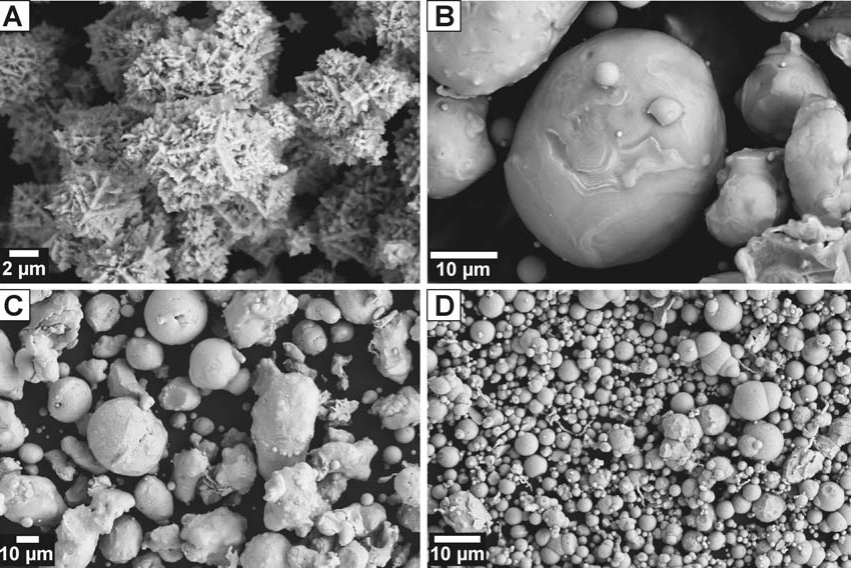
SEM photographs of unreacted alloys/metals used in this study. A) Ni metal, b) Ni_81_Fe_19_, c) Ni_50_Fe_50 _and d) Fe metal.

Metals and alloys representing an fcc solid solution of Ni in Fe were purchased from Alfa Aesar^® ^and Goodfellow. All starting and selected reacted solids were characterized by X-Ray Diffractometry, X-Ray Photoelectron Spectroscopy, Scanning Electron Microscopy, B.E.T. surface analysis, and Electron Microprobe. The results of metal/alloy characterization are summarized in Tab. 2 and Fig. 2, 3.

**Table 2:** Conditions and results of reduction experiments conducted in this study. All concentrations are in μmol.kg^-1^. Values and their errors bigger than 10 are rounded to the nearest whole number, those smaller than 10 are rounded to the nearest tenth.

***Metal/Alloy***	***Run***	***Solution***	***T°C***	***pH*_*pre*_**	***pH*_*post*_**	***NH*_4_^+^**	***%***	*NH*_4_^+^_norm_*	*NO*_2_^-**^	*NO*_3_^-**^
Fe	1	Ar	200	5.7	9.9	171 ± 1	-	340 ± 21	n.a.	n.a.
Fe	2	650 N_2_	200	5.8	8.9	187 ± 1	2.5	371 ± 23	n.a.	n.a.
Fe	3	39/617 H_2_/N_2_	200	5.8	8.4	230 ± 1	10	455 ± 28	n.a.	n.a.
Fe	4	459 KCl in N_2_	200	6.2	8.9	267 ± 2	15	529 ± 32	n.a.	n.a.
Fe	5	484 KNO_2 _in N_2_	200	6.0	9.8	768 ± 1	100	1522 ± 93	0	0
Fe	6	465 KNO_3 _in N_2_	200	6.2	9.8	760 ± 8	100	1507 ± 92	0	0
Fe	7	485 KNO_2 _in Ar	200	6.0	10.2	749	100	1486 ± 91	0	0
Fe	8	477 KNO_3 _in Ar	200	6.2	10.3	752 ± 3	100	1491 ± 91	0	0
Ni	9	Ar	200	5.8	8.7	18	-	27 ± 3	n.a.	n.a.
Ni	10	650 N_2_	200	5.7	8.3	28	1.5	43 ± 5	n.a.	n.a.
Ni	11	39/617 H_2_/N_2_	200	5.8	6.3	30	1.8	45 ± 5	n.a.	n.a.
Ni	12	459 KCl in N_2_	200	6.2	6.7	20	0.3	30 ± 4	n.a.	n.a.
Ni	13	484 KNO_2 _in N_2_	200	6.0	9.1	559 ± 8	100	855 ± 102	0	0
Ni	14	465 KNO_3 _in N_2_	200	6.2	9.0	538 ± 7	100	823 ± 98	0	0
Ni	15	485 KNO_2 _in Ar	200	6.0	9.3	544 ± 7	100	833 ± 99	0	0
Ni	16	477 KNO_3 _in Ar	200	6.2	9.3	543 ± 4	100	830 ± 99	0	0
Ni_50_Fe_50_	17	Ar	200	5.8	8.7	15	-	40 ± 1	n.a.	n.a.
Ni_50_Fe_50_	18	650 N_2_	200	5.7	7.9	17	0.3	47 ± 1	n.a.	n.a.
Ni_50_Fe_50_	19	39/617 H_2_/N_2_	200	5.6	8	17	0.3	45 ± 1	n.a.	n.a.
Ni_50_Fe_50_	20	476 KCl in N_2_	200	6.3	7.1	14	0	39 ± 1	n.a.	n.a.
Ni_50_Fe_50_	21	480 KNO_2 _in N_2_	200	5.9	9.8	418 ± 3	100	1133 ± 29	0	0
Ni_50_Fe_50_	22	463 KNO_3 _in N_2_	200	6.0	9.7	490 ± 2	100	1330 ± 34	0	0
Ni_50_Fe_50_	23	474 KNO_2 _in Ar	200	6.1	10.3	475 ± 5	100	1289 ± 33	0	0
Ni_50_Fe_50_	24	487 KNO_3 _in Ar	200	6.0	9.9	476 ± 6	100	1293 ± 33	0	0
Ni_81_Fe_19_	25	Ar	200	5.8	8.6	18	-	98 ± 7	n.a.	n.a.
Ni_81_Fe_19_	26	650 N_2_	200	5.7	7.9	19	0.2	99 ± 7	n.a.	n.a.
Ni_81_Fe_19_	27	39/617 H_2_/N_2_	200	5.6	8.3	19	0.2	103 ± 7	n.a.	n.a.
Ni_81_Fe_19_	28	476 KCl in N_2_	200	6.3	6.2	18 ± 1	0	96 ± 7	n.a.	n.a.
Ni_81_Fe_19_	29	480 KNO_2 _in N_2_	200	5.9	9.9	410 ± 6	100	2186 ± 153	0	0
Ni_81_Fe_19_	30	463 KNO_3 _in N_2_	200	6.0	9.8	483 ± 4	100	2576 ± 180	0	0
Ni_81_Fe_19_	31	474 KNO_2 _in Ar	200	6.1	10.3	485 ± 4	100	2583 ± 180	0	0
Ni_81_Fe_19_	32	487 KNO_3 _in Ar	200	6.0	9.9	493 ± 3	100	2626 ± 183	0	0
-	33	497 KNO_2_	200	6.0	9.4	45	9.1	-	288 ± 2	73 ± 1
-	34	444 KNO_3_	200	6.1	6.4	3.6	0.8	-	273 ± 1	129 ± 1
-	35	506 FeCl_2 _in N_2_	200	4.7	3.3	8.1 ± 0.1	1.2	-	n.a.	n.a.
-	36	492 NiCl_2 _in N_2_	200	5.3	5.7	8.0 ± 0.3	1.2	-	n.a.	n.a.
Fe	37	Ar	70	5.7	7.6	3.8	-	7.4 ± 0.5	n.a.	n.a.
Fe	38	650 N_2_	70	5.8	8.5	3.6	0	7.2 ± 0.4	n.a.	n.a.
Fe	39	492 KNO_2 _in O_2_	70	6.9	10.1	559 ± 3	100	1107 ± 68	0	0
Fe	40	502 KNO_2 _in N_2_	70	6.1	9.8	550 ± 3	100	1091 ± 67	0	0
Fe	41	511 KNO_3 _in O_2_	70	6.7	9.9	558 ± 2	98	1106 ± 68	0	8.8 ± 0.2
Fe	42	486 KNO_3 _in N_2_	70	6.3	10.1	512 ± 6	98	1015 ± 62	0	9.6 ± 0.2
Ni	43	Ar	70	5.7	7.6	0.9	-	1.4 ± 0.2	n.a.	n.a.
Ni	44	650 N_2_	70	5.8	6.9	0.9	0	1.4 ± 0.2	n.a.	n.a.
Ni	45	492 KNO_2 _in O_2_	70	6.9	10.1	528 ± 2	100	807 ± 96	0	0
Ni	46	502 KNO_2 _in N_2_	70	6.1	9.9	534 ± 3	100	818 ± 97	0	0
Ni	47	511 KNO_3 _in O_2_	70	6.7	10.2	521 ± 5	100	797 ± 95	0	0
Ni	48	486 KNO_3 _in N_2_	70	6.3	10.2	534 ± 5	100	817 ± 97	0	0
Ni_50_Fe_50_	49	Ar	70	5.7	8.2	0.4	-	1.2	n.a.	n.a.
Ni_50_Fe_50_	50	650 N_2_	70	5.8	8.3	0.4	0	1.2	n.a.	n.a.
Ni_50_Fe_50_	50	492 KNO_2 _in O_2_	70	6.9	8.4	22 ± 1	3.9	61 ± 2	473 ± 3	0
Ni_50_Fe_50_	51	502 KNO_2 _in N_2_	70	6.1	8.1	23 ± 1	2.8	62 ± 2	488 ± 2	0
Ni_50_Fe_50_	52	511 KNO_3 _in O_2_	70	6.7	8.0	19 ± 1	8	53 ± 1	0	470 ± 2
Ni_50_Fe_50_	53	486 KNO_3 _in N_2_	70	6.3	8.4	28 ± 1	6.2	77 ± 2	0	456 ± 2
Ni_81_Fe_19_	54	Ar	70	5.7	8.2	0.5	-	2.9 ± 0.2	n.a.	n.a.
Ni_81_Fe_19_	55	650 N_2_	70	5.8	8.1	0.5	0	2.7 ± 0.2	n.a.	n.a.
Ni_81_Fe_19_	56	492 KNO_2 _in O_2_	70	6.9	9.2	23 ± 1	4.7	125 ± 9	410 ± 1	0
Ni_81_Fe_19_	57	502 KNO_2 _in N_2_	70	6.1	9.2	71 ± 2	14	379.27	406 ± 1	0
Ni_81_Fe_19_	58	511 KNO_3 _in O_2_	70	6.7	8.6	35	6.8	186 ± 13	13 ± 1	425 ± 2
Ni_81_Fe_19_	59	486 KNO_3 _in N_2_	70	6.3	8.7	69 ± 2	14.2	370 ± 26	9.1 ± 0.7	424 ± 3
Fe	60	Ar	22	5.7	6.4	0.7	-	1.4	n.a.	n.a.
Fe	61	650 N_2_	22	5.8	6.6	0.7	0	1.4	n.a.	n.a.
Fe	62	541KNO_2 _in O_2_	22	6.2	10.8	544 ± 6	100	1078 ± 66	0	0
Fe	63	549 KNO_2 _in N_2_	22	6.0	10.6	542 ± 5	99	1075 ± 66	0	0
Fe	64	529 KNO_3 _in O_2_	22	6.4	10.5	527 ± 4	100	1044 ± 64	0	0
Fe	65	521 KNO_3 _in N_2_	22	5.9	10.8	486 ± 5	93	963 ± 59	0	0
Ni	66	Ar	22	5.7	6.9	0.4	-	0.6	n.a.	n.a.
Ni	67	650 N_2_	22	5.8	5.6	0.4	0	0.6	n.a.	n.a.
Ni	68	541KNO_2 _in O_2_	22	6.2	10.6	422 ± 3	78	646 ± 77	0	0
Ni	69	549 KNO_2 _in N_2_	22	6.0	10.7	451 ± 5	82	690 ± 82	0	0
Ni	70	529 KNO_3 _in O_2_	22	6.4	10.2	139	26	213 ± 25	43	36
Ni	71	521 KNO_3 _in N_2_	22	5.9	10.1	364 ± 1	70	557 ± 66	1.2	22
Ni_50_Fe_50_	72	Ar	22	5.7	6.3	0	-	0	n.a.	n.a.
Ni_50_Fe_50_	73	650 N_2_	22	5.8	6.3	0	0	0	n.a.	n.a.
Ni_50_Fe_50_	74	541 KNO_2 _in O_2_	22	6.2	6.8	1.4	0.3	3.9 ± 0.1	553 ± 2	0
Ni_50_Fe_50_	75	549 KNO_2 _in N_2_	22	6.0	7.7	1.1 ± 0.1	0.2	3.1 ± 0.1	556	0
Ni_50_Fe_50_	76	529 KNO_3 _in O_2_	22	6.4	7.2	0.8	0.2	2.1 ± 0.1	0	526 ± 1
Ni_50_Fe_50_	77	521 KNO_3 _in N_2_	22	5.9	7.3	1	0.2	2.7 ± 0.1	0	523 ± 2
Ni_81_Fe_19_	78	Ar	22	5.7	6.9	0	-	0	n.a.	n.a.
Ni_81_Fe_19_	79	650 N_2_	22	5.8	6.6	0	0	0	n.a.	n.a.
Ni_81_Fe_19_	80	541 KNO_2 _in O_2_	22	6.2	9.4	3 ± 0.1	0.6	16 ± 1	515 ± 2	0
Ni_81_Fe_19_	81	549 KNO_2 _in N_2_	22	6.0	9.3	1.9 ± 0.1	0.4	10 ± 1	539 ± 3	0
Ni_81_Fe_19_	82	529 KNO_3 _in O_2_	22	6.4	8.9	0	0	0	0	493 ± 1
Ni_81_Fe_19_	83	521 KNO_3 _in N_2_	22	5.9	9.0	1.9 ± 0.1	0.4	10 ± 1	0	482

"n.a." denotes "not analyzed, "-" not applicable. * denotes NH_4_^+ ^concentration formed in the experiment normalized to 1 m^2 ^of surface area of the metal/alloy. ** denote residual concentration after the experiment was completed.

X-Ray Diffraction (XRD) data were collected using a Scintag PAD X diffractometer under the following conditions: CuK_α1_, 40 kV, 25 mA, 5° – 90° 2Θ, step 0.02° 2Θ and variable scan rates.

Scanning Electron Analysis (SEM) was performed on the LEO 1550 SFEG scanning electron microscope equipped with an EDAX energy dispersive X-ray spectrometer (EDS) using an accelerating voltage of 15 kV and a 30 μm aperture.

Oxidation state of Ni, Fe and the presence of N in alloys were determined by X-Ray Photoelectron Spectroscopy (XPS). The data were acquired with unmonochromatized Mg_Kα _and Al_Kα _radiation at 1253.6 eV and 1486.7 eV using a Physical Electronics source controller in a vacuum chamber with a base pressure of 1 × 10^-9 ^Torr. A VG Microtech hemispherical analyzer was used to obtain the energy distribution of the photoemitted electrons at pass energies of 50 and 75 eV. The binding energies were calibrated by fixing the Au 4p3/2 and 4f7/2 peaks (546.3 eV, 87.5 eV) from a gold standard, and the metallic Fe 2p3/2 and Ni 2p3/2 cores (707.0 eV, 852.3 eV). Selected particles were sputtered with Ar^+ ^accelerated to 2 kV to expose their interior and check for the presence of nitrogen using a Physical Electronics ion gun controller.

B.E.T. surface analysis (BET) was performed using a Micromeritics ASAP 2010 analyzer with a 10-mm Hg transducer using UHP N_2 _gas. The surface area was calculated from measurements at 5 different N_2_(g) pressures (42.58196, 85.30770, 132.82384, 180.25029 and 227.66101 Torr).

Chemical composition of alloys/metals was determined by a Cameca Camebax Micro electron microprobe (EPMA) equipped with four wavelength dispersive spectrometers and a Kevex Analyst 8000 energy dispersive detector. During all analyses, the accelerating voltage and beam current used were 15 kV and 10 nA (nominal), respectively.

Molecular hydrogen was analyzed on a SRI 8610C single column gas chromatograph (GC) with a TCD detector, 6' Hayesep D column and N_2 _carrier gas. The sample (~0.2 mL) was withdrawn from the reaction vessel into a gastight^® ^Hamilton™ syringe with a Mininert™ valve and immediately analyzed using a 4-point calibration curve. Gas mixtures (Matheson™) of known composition were used as calibration standards.

Total nitrogen content of metals/alloys was analyzed by IMR Test Labs (Lansing, NY) by inert gas fusion [35]. During the analysis, N is released from the metal at 1900°C into the stream of He gas and analyzed in a thermal conductivity cell.

Experimental solutions were analyzed for NH_4_^+ ^using a Dionex DX-500 ion chromatograph (IC) with a 100 μL sample loop. NH_4_^+ ^and NO_3_^-^/NO_2_^- ^were analyzed using a 4 mm Dionex IonPac^® ^CS-16 (40°C, 34 mN H_2_SO_4 _eluent) and IonPac^® ^AS4A-SC (22°C, 5 mmol.kg^-1 ^Na_2_B_4_O_7 _eluent), respectively. Concentrations were calculated from a 4-point calibration curve with R^2 ^values above 0.99.

No other compounds were analyzed. It is expected, however, that other reaction products and/or intermediates (e.g., NO) may have formed during a complex sequence of electron transfer reactions.

Geochemical equilibrium modeling was performed with the Geochemist's Workbench 5 [36] software package with the thermo.com.V8.R6.full thermodynamic database complemented with data for Ni_3_Fe and NiFe [37].

## Results

### Dinitrogen reduction

**Figure 4:**
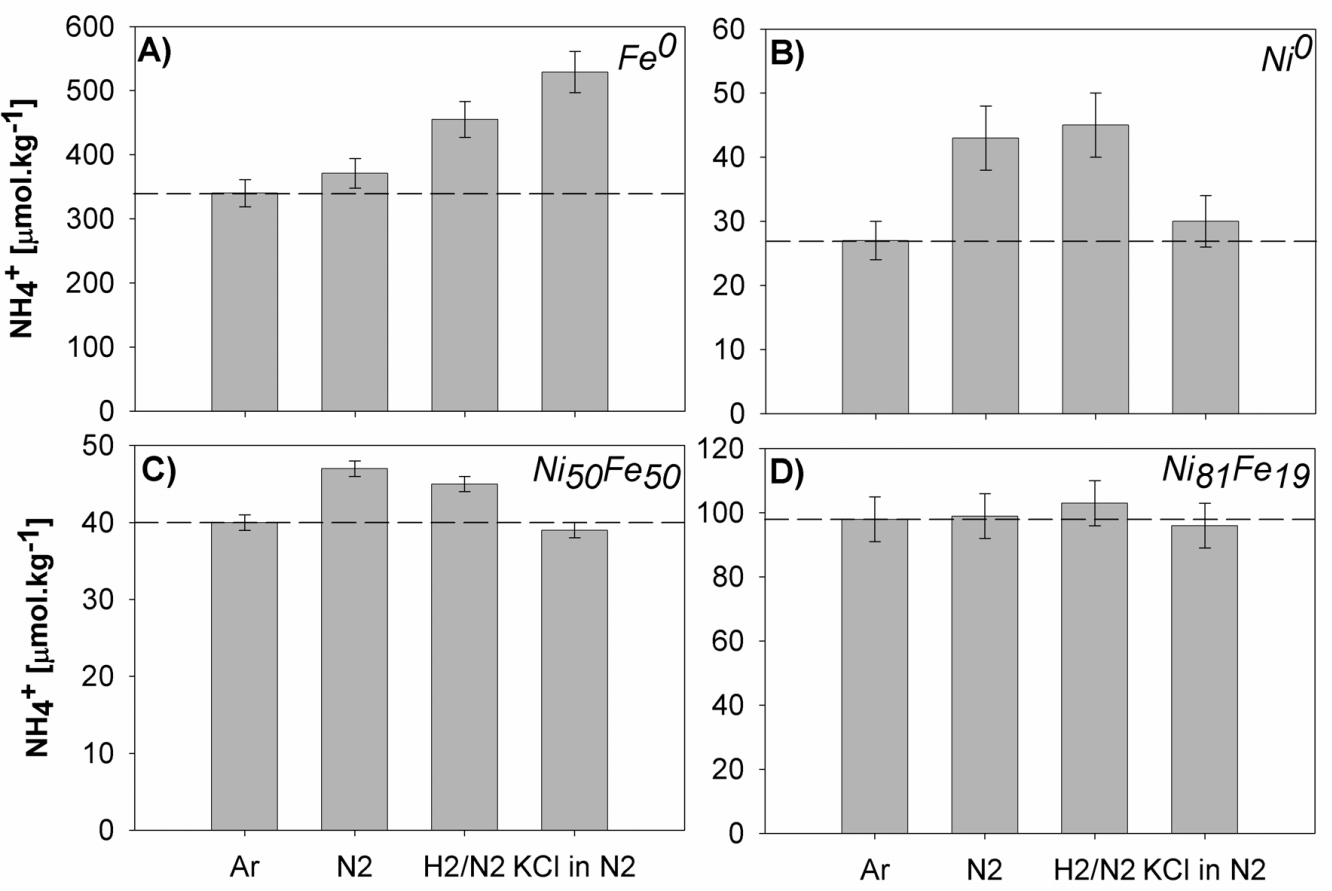
NH_4_^+ ^formation from N_2 _in the presence of Ni, Fe metals and alloys at 200°C. The dashed lines correspond to the Ar blank. The concentrations of KCl in the experiments were 459 μmol.kg^-1 ^in A, B and 476 μmol.kg^-1 ^in C, D. Results are normalized to 1 m^2 ^of surface area.

The results of NH_4_^+ ^formation from N_2 _and the effect of added H_2 _and KCl at 200°C (normalized to 1 m^2 ^surface area) are shown in Fig. 4 and summarized in Tab. 1 (runs 1–32). All results are compared with respect to blank experiments conducted with Ar and no N_2 _added. The blank experiments thus represent the background NH_4_^+ ^production from the metal/alloy involved and reactor catalysis. Error bars were calculated by propagating errors from solution dilutions and B.E.T. and IC analyses. Due to relatively large error bars, only results differing from the blank (or each other) by more than the calculated error will be discussed.

In the presence of N_2_, only Ni_81_Fe_19 _did not show appreciable activity toward NH_4_^+ ^formation (Fig. 4d). Within our experimental certainty, Fe^0 ^was the material associated with the most NH_4_^+ ^production (31 μmol.kg^-1^.m^2^, Fig. 4a), followed by Ni^0 ^(16 μmol.kg^-1^.m^2^, Fig. 4b) and Ni_50_Fe_50 _(7 μmol.kg^-1^.m^2^, Fig. 4c). H_2_(aq) was observed to form in the presence of all studied metals and alloys. Representative concentrations of H_2_(aq) at 200°C (measured in one experiment with N_2_-saturated, O_2_-free DI per metal/alloy only) were 0.38 mmol.kg^-1 ^with Ni^0^, 0.28 mmol.kg^-1 ^with Ni_81_Fe_19_, 0.7 mmol.kg^-1 ^with Ni_50_Fe_50 _and 21 mmol.kg^-1 ^with Fe^0^. We point out that this H_2 _production is relatively large compared to the background H_2 _production of the Ti reaction vessels that was determined to be 12 μmol.kg^-1 ^in an experiment with DI saturated with Ar at 200°C. An interesting observation during our experiments was that the reactivity of Fe^0 ^towards O_2_-free DI was so rapid that gas bubbles were forming on its surface after just a few hours of exposure at 22°C (by analogy with 200°C experiments we assume it is H_2_).

Our results showed that the addition of H_2 _(5%/95% H_2_/N_2_) into the reaction mixture only resulted in change in the Fe^0 ^circumstance where 158 μmol.kg^-1^.m^2 ^NH_4_^+ ^was produced (Fig. 4a). Fe^0 ^was also the material most affected by the addition of K^+ ^(KCl). In this case, 189 μmol.kg^-1^.m^2 ^of NH_4_^+ ^was produced, 158 μmol.kg^-1^.m^2 ^more than with N_2 _alone (Fig. 4a). In general, our experiments showed that Ni_81_Fe_19 _was the least, and Fe^0^, the most, affected by the addition of H_2 _or KCl into the reacting solution (Fig. 4d, a).

Aqueous Fe and Ni cations (NiCl_2_, FeCl_2_) only had a small effect on N_2 _reduction chemistry, converting about 1% of the total available N into NH_4_^+ ^at 200°C (Tab. 1). A post-reaction visual inspection of the FeCl_2 _solution showed a fine-grained colloid of reddish color that, in the presence of atmospheric O_2 _changed color to light brown. The amount of recovered solids was insufficient for analysis by XRD.

At temperatures of 70 and 22°C no NH_4_^+ ^formation from N_2 _was observed (Tab 1, runs 38, 44, 50, 55, 61, 67, 73, 79). Use of these lower temperatures also resulted in lower background NH_4_^+ ^production in solutions containing the metals/alloys. The yield of NH_4_^+ ^ranged from 7.4 (Fe^0^) to 1.2 μmol.kg^-1^.m^2 ^(Ni_50_Fe_50_) at 70°C (Tab. 1, runs 37, 43, 49, 54) and 1.4 (Fe^0^) to 0 μmol.kg^-1^.m^2 ^(Ni_50_Fe_50_, Ni_81_Fe_19_) at 22°C (Tab. 1, runs 60, 66, 72, 78).

### Nitrite and nitrate reduction

**Figure 5:**
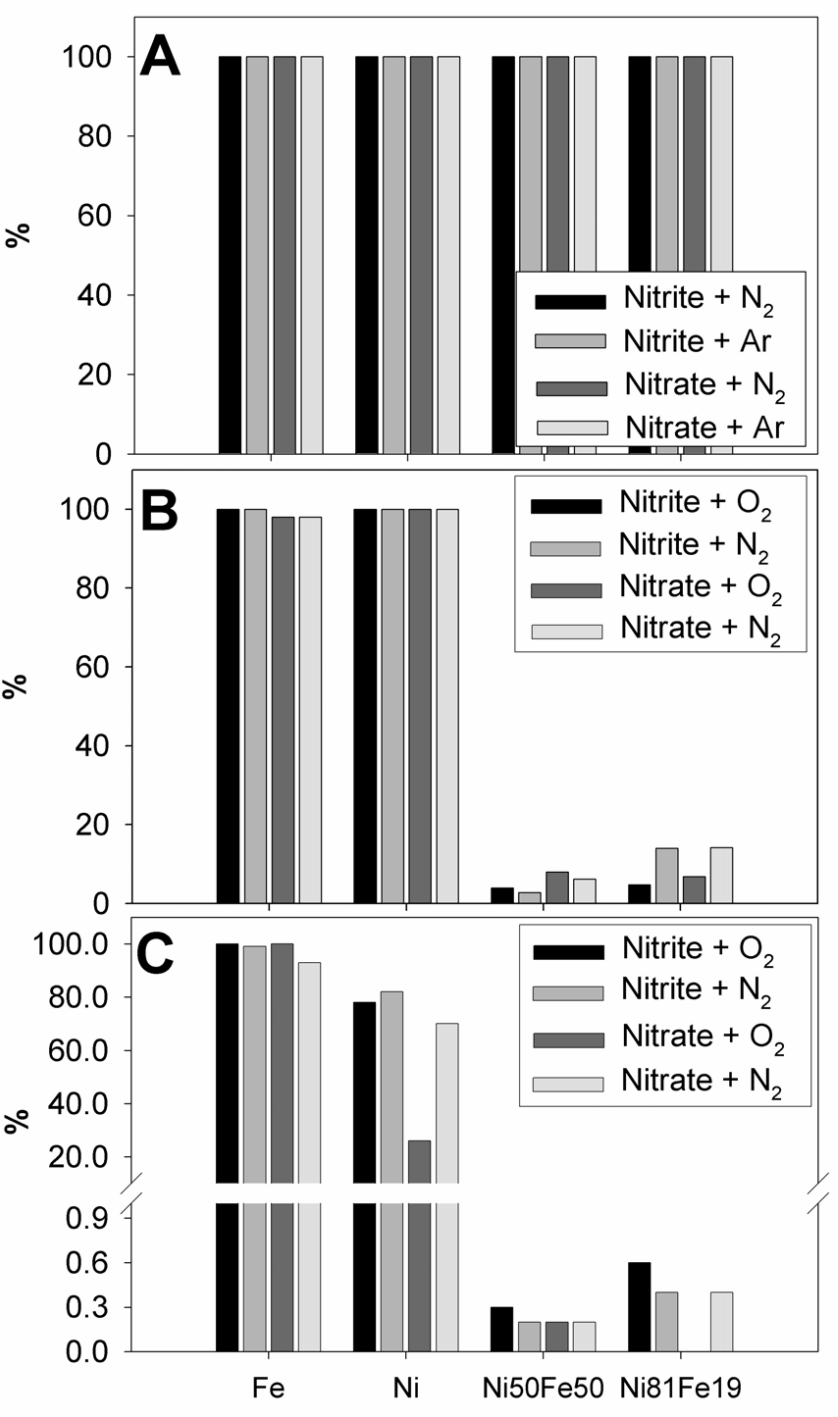
NH_4_^+ ^formation from nitrite and nitrate, expressed in terms of % conversion. Panels A, B and C correspond to sets of experiments at 200, 70 and 22°C, respectively.

The results of our NO_2_^- ^and NO_3_^- ^reduction experiments are shown in Fig. 5. At 200°C all tested metals/alloys were found to be very effective in converting NO_2_^-^/NO_3_^- ^into NH_4_^+^. To assess if the presence of NO_2_^- ^or NO_3_^- ^in the solution had any effect on N_2 _reduction, each experiment was conducted in duplicate with Ar (Tab. 1, runs 7, 8, 15, 16, 23, 24, 31, 32) and N_2 _(Tab. 1, runs 5, 6, 13, 14, 21, 22, 29, 30) saturated solutions (e.g., Fe^0 ^with NO_2_^-^/N_2 _and NO_2_^-^/Ar). These experiments taken in sum showed that there was no difference between the N_2 _and Ar runs (Fig. 5a).

The results collected at 70°C reveal prominent differences between alloys and metals (Fig. 5b). While Ni and Fe achieved almost 100% conversions of NO_2_^-^/NO_3_^- ^to NH_4_^+ ^(Tab. 1, runs 40, 42, 46, 48), interestingly, no- or insignificant reduction (less than 20%) was observed in solutions reacted with alloys (Tab. 1, runs 51, 53, 57, 59). The presence of O_2 _in the solution appears to have an inhibitive effect on the reduction process, especially in the presence of Ni_81_Fe_19 _alloy (Tab. 1, runs 56, 58; Fig. 5b).

Low temperature experiments (22°C) further confirmed the temperature dependence of NO_2_^-^/NO_3_^- ^reduction in the presence of alloys (Fig. 5c). Compared to 200°C and even at 70°C, NH_4_^+ ^formation was negligible (less than 1% conversion) (runs 63, 65, 69, 71, 75, 77, 81, 83). The effect of O_2 _in the reacted solution was most pronounced with NO_3_^- ^in the presence of Ni^0 ^(run 70). In general, at 22°C Fe^0 ^was the most efficient material in converting NO_2_^-^/NO_3_^- ^to NH_4_^+^, regardless of the O_2 _content (Tab. 1, runs 62–65; Fig. 5c).

It is important to note that at 200°C both NO_2_^- ^and NO_3_^- ^decomposed in the absence of metal/alloys as well (Tab. 1, runs 34, 34). 42% of the initial 497 μmol.kg^-1 ^KNO_2 _solution was converted into NO_3_^- ^(~15%), NH_4_^+ ^(~9%) and other N compounds (~18%) that were not analyzed. Of the initial 444 μmol.kg^-1 ^KNO_3 _solution, 71% was converted into NO_2_^- ^(~61%), NH_4_^+ ^(~1%) and about 9% corresponds to other unanalyzed N compounds. It is not clear if this is a result of thermally induced decomposition, catalysis or reaction by/with the titanium reaction vessel, or a combination of all; nevertheless, NH_4_^+ ^was not the dominant reaction product. At 70 and 22°C, both NO_2_^- ^and NO_3_^- ^solutions were found to be stable in experiments without metals or alloys during the 24-hour reaction period.

### Metal/alloy alteration

**Figure 6:**
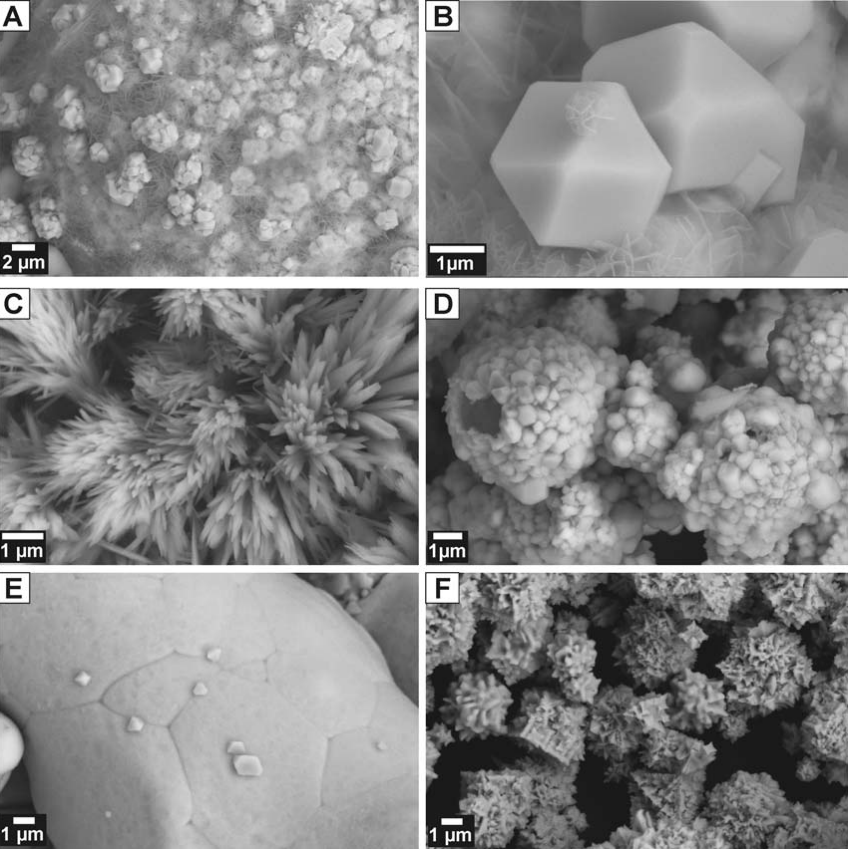
SEM microphotographs of reacted metals and alloys. A) Fe^0 ^reacted in N_2_(aq) solution depicting coexisting magnetite and Fe-(oxy)hydroxides; B) Detail of magnetite single crystals formed on Fe^0^; C) clusters of needle-like Fe-(oxy)hydroxide crystals formed on Fe^0^; D) pseudomorphoses of magnetite after Fe^0 ^in the H_2_/N_2 _solution; E) magnetite crystals formed on Ni_50_Fe_50 _in the KNO_3 _solution; F) Ni^0 ^skeletal crystal revealing no change after reaction.

**Figure 7:**
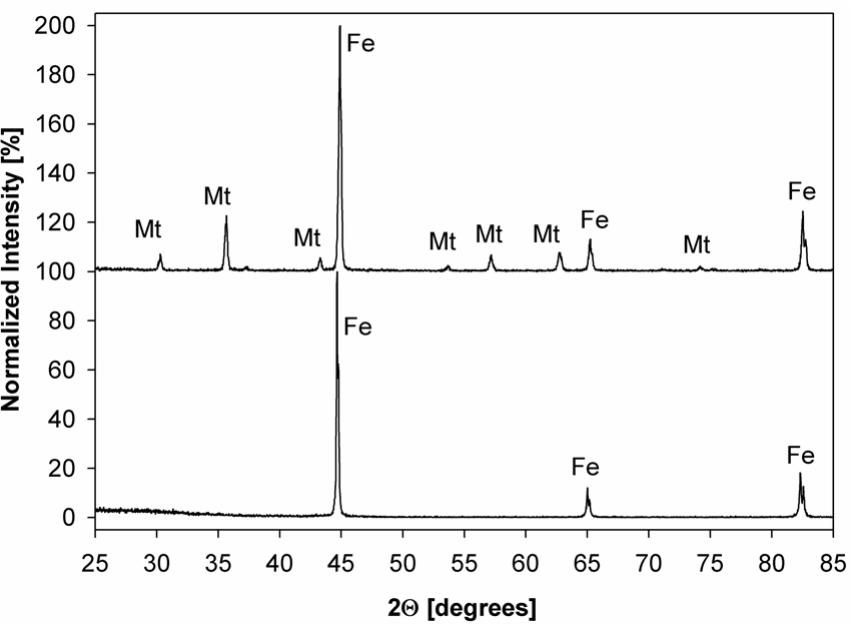
Normalized XRD patterns of unreacted (lower) reacted (upper) Fe^0 ^showing the presence of magnetite (Mt).

**Figure 8:**
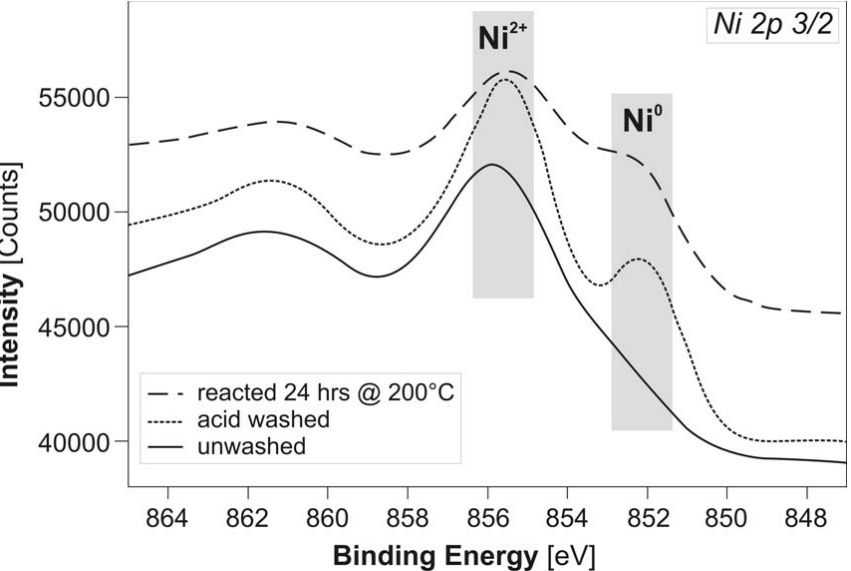
Ni 2p3/2 peaks showing the speciation of Ni on the surface Ni metal powder at various stages of the experiment. Note the presence of residual zerovalent Ni on the surface after 24 hour reaction at 200°C.

Generally, the extent of alteration of Fe-containing metal/alloy increased with temperature, as demonstrated by the presence of secondary minerals (Fig. 6). Magnetite (Fe_3_O_4_) was the most abundant alteration product, predominantly forming euhedral to subhedral crystals up to several μm in size (Fig. 6a, b, d, e). Pseudomorphoses of magnetite after reacted spherical Fe^0 ^particles were common (Fig. 6d). The second most common alteration phase were Fe-(oxy)hydroxides (e.g., lepidocrocite) usually of amorphous appearance or forming needle-like (Fig. 6c) and platy crystals several tens of nm thin and several μm long. Both magnetite and Fe-(oxy)hydroxides commonly occur simultaneously in all reacted samples (SEM) (Fig. 6a,b); however, only magnetite is identified by the XRD method (Fig. 7). This suggests that the Fe-(oxy)hydroxides either lack long range order (e.g., "X-ray amorphous") and/or their abundance is less than 5%, the approximate detection limit of XRD. In general, the lower the Fe content, the lower the extent of alteration (Fig. 6e). In contrast, reacted Ni^0 ^exhibited no microscale (SEM) evidence of reaction (Fig. 6f, compare with Fig. 3a), as corroborated by the XPS spectra documenting the presence of residual zero-valent Ni species on the surface (Fig. 8). As a result of solution interactions with metals/alloys, the resulting pH in most experiments was higher than the starting value (see discussion) (Tab. 1).

## Discussion

### Nitrogen in alloys and metals

**Figure 9:**
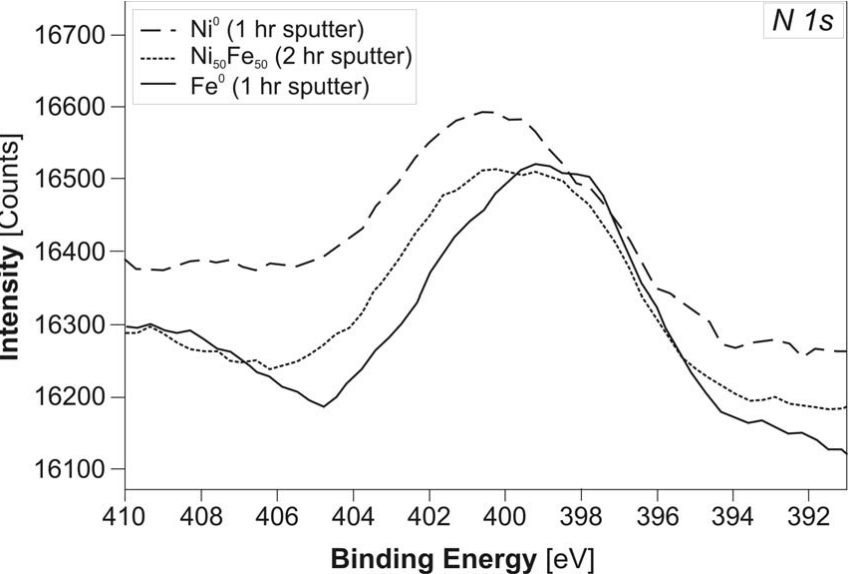
N 1 s peaks centered around 400 eV documenting the presence of N in the tested alloys and metals after sputtering with Ar^+ ^(Ni^0 ^400.1 eV, Ni_50_Fe_50 _399.7 eV, Fe^0 ^398.9 eV).

All the metals and alloys investigated in this work were found to contain N, which resulted in a background production of NH_4_^+^. The presence of atomic N was based on two lines of evidence: 1) The presence of the N1s peak in the XPS spectra of starting metals and alloys even after "cleaning" the surface by sputtering with Ar^+ ^ions (Fig. 9) and; 2) quantitative analysis of N_tot _content of the starting materials by inert gas fusion with a thermal conductivity detection (Tab. 3).

**Table 3:** Total N content of metals and alloys used in this study as determined by inert gas fusion – thermal conductivity method (IMR Test Labs).

**Sample**	**N_TOTAL _[wt.%]**
Fe^0^	0.0124 ± 0.001
Ni^0^	0.0012 ± 0.0005
Ni_50_Fe_50_	0.0030 ± 0.0005
Ni_81_Fe_19_	0.0009 ± 0.0005

Commercially available Ni, Fe metals/alloys may contain N due to the manufacturing process which employs either inert (N_2_, Ar) or reducing (NH_3_, H_2_) atmospheres to prevent oxidation [38,39] [Alfa Aesar, Goodfellow – pers.comm]. For example, during the synthesis, N_2 _chemisorbs on the surface of molten/hot metal and dissociates (Equation 1).

(1) N_2 _(gas) → 2N (in metal)

In the subsequent step N enters the structure via diffusion or convection to form primarily monoatomic interstitial and to lesser extent substitutional solid solutions. This process is governed by the Sievert's law (Equation 2), which predicts that diatomic gases such as N_2 _dissolve in metals (*c*_*N*_) proportionally to the square root of the partial pressure (*p*_*N*2_) in the coexisting gas phase [40,41].

(2) $$K=\frac{c_{N}^{2}}{p_{N_{2}}}$$

Assuming homolytic N_2 _bond cleavage, each N atom would have three unpaired electrons available for bonding with the surrounding metal atoms. XPS spectra collected from unreacted metals/alloys in our experiments point to nitride (N^3-^, 398.6 eV) as the likely N species (Fig. 9) [42]. Consequently, upon release into the solution, N^3- ^is expected to react with available protons to form NH_3_/NH_4_^+ ^(Reaction 3, 4) and contribute to their high background productivity.

(3) N^3- ^+ 3H^+ ^→ NH_3_

(4) N^3- ^+ 4H^+ ^→ NH_4_^+^

However, undissociated N_2 _gas may get trapped in the molten metal (e.g., in inclusions) as well.

### Dinitrogen reduction

Although batch-type experiments, such as the ones described above, provide little insight into the kinetics of a reaction and much less the reaction mechanism, the results do allow one to compare the amount of NH_3_/NH_4_^+ ^formed under different conditions. In addition the results can be placed in the context of previously published research related to the reduction of N-species to NH_3_/NH_4_^+^. Due to the immense importance of NH_3_/NH_4_^+ ^in industry and agriculture, several decades of research exist on its synthesis and production from N_2 _gas [e.g., [43]]. The industrial Haber-Bosch process utilizes Fe^0 ^catalyst at high temperatures and pressures (~500°C, ~100 bars) to synthesize NH_3 _from H_2 _and N_2 _gas (Reaction 5).

(5) N_2_(g) + 3H_2_(g) → 2NH_3_(g)

In brief, the reaction proceeds as follows: sorption of H_2 _and N_2 _gases on the surface is followed by the formation of atomic H_ads _and N_ads _(dissociative sorption). Fe^0 ^then facilitates electron transfer from H_ads _to N_ads _(e.g., N reduction), followed by the formation of NH_3 _gas on the surface and subsequent desorption. The dissociative chemisorption of N_2 _is generally taken to be the rate-limiting step [44]. For comparison, modern life overcomes the reaction's activation barrier using the enzyme *nitrogenase *composed of dinitrogenase (MoFe center) and dinitrogenase reductase (Fe center) proteins. In Reaction 6, Fd stands for ferredoxin, the electron-transfer protein [45,46].

(6) N_2_(g) + 8Fd^0^_red _+ 10H^+ ^→ 2NH_4_^+ ^+ 8Fd^+^_ox _+ H_2_(g)

Analogous overall reactions of abiotic N_2 _reduction can be written for aqueous solutions (Reaction 7, 8), although it is important to note that since this reaction requires a transfer of multiple electrons, several reaction intermediates must be involved. Once in solution, NH_3 _and NH_4_^+ ^exist in a pH dependent equilibrium (Equation 9; valid for 25°C).

(7) N_2_(aq) + 2H^+ ^+ 3H_2_(aq) → 2NH_4_^+^

(8) N_2_(aq) + 3H_2_(aq) → 2NH_3_(aq)

(9) $$pH=9.25+\log\left\{\frac{\left[NH_{3}\right]}{\left[NH_{4}^{+}\right]}\right\}$$

We hypothesize that reactions between H_2_, N_2 _and the metal/alloy surface are taking place in our experiments; however, they are orders of magnitude slower than those described above in the gas phase. Undoubtedly, this is due to the fact that aqueous reactions occurring in our experiments are not only more complex but also take place at much lower *T*, *P *conditions and H_2_/N_2 _concentrations than those typical for Haber-Bosch synthesis. Such kinetic constraints could explain low NH_4_^+ ^yields, even though the thermodynamic equilibrium models predict NH_4_^+ ^to be the dominant N species (Tab. 4).

**Table 4:** Predicted equilibrium pH and nitrogen speciation in the N-H_2_O-Fe-Ni system (only species with molalities above 1·10^-8 ^shown). Concentrations and fugacities of dominant N species are in bold. The last column shows the predicted final mineral assemblage.

***Mineral***	***T [°C]***	***pH***	***mNH*_3_**	***mNH*_4_^+^**	***mN*_2_**	***mH*_2_**	***fNH*_3_**	***fN*_2_**	***fH*_2_**	***Assemblage***
Nickel	200	6.95	**6.4·10**^-4^	4.63·10^-5^	2.47·10^-4^	3.73·10^-5^	1.24·10^-3^	**0.2769**	0.24	BN, NI
Nickel	70	8.92	**1.00·10**^-3^	1.34·10^-4^	2.2·10^-5^	2.68·10^-7^	9.98·10^-5^	**0.0477**	3.68·10^-4^	BN, NI
Nickel	22	10.2	**1.03·10**^-3^	1.34·10^-4^	8.12·10^-6^	2.18·10^-8^	1.41·10^-5^	**0.0118**	2.71·10^-5^	BN, NI
Awaruite	200	7.07	**1.12·10**^-3^	6.14·10^-5^	<1·10^-8^	0.0597	**2.16·10**^-3^	<1·10^-8^	38.54	AW, MT, NI
Awaruite	70	8.93	**1.04·10**^-3^	1.37·10^-4^	<1·10^-8^	0.0152	**1.04·10**^-4^	<1·10^-8^	20.85	AW, MT, NI
Awaruite	22	10.2	**1.05·10**^-3^	1.35·10^-4^	<1·10^-8^	0.0103	**1.44·10**^-5^	<1·10^-8^	12.82	AW, MT, NI
Tetrataenite	200	8.7	**5.92·10**^-4^	7.96·10^-7^	<1·10^-8^	0.0865	**1.14·10**^-3^	<1·10^-8^	55.85	AW, MT, TT
Tetrataenite	70	8.93	**1.04·10**^-3^	1.37·10^-4^	<1·10^-8^	0.0222	**1.04·10**^-4^	<1·10^-8^	30.51	AW, MT, TT
Tetrataenite	22	10.2	**1.05·10**^-3^	1.35·10^-4^	<1·10^-8^	0.0159	**1.44·10**^-5^	<1·10^-8^	19.73	AW, MT, TT
Iron	200	7.07	**1.15·10**^-3^	6.22·10^-5^	<1·10^-8^	1.532	**2.22·10**^-3^	<1·10^-8^	989.1	MT, FE
Iron	70	8.93	**1.06·10**^-3^	1.38·10^-4^	<1·10^-8^	0.785	**1.05·10**^-4^	<1·10^-8^	1077	MT, FE
Iron	22	10.2	**1.06·10**^-3^	1.36·10^-4^	<1·10^-8^	0.623	**1.45·10**^-5^	<1·10^-8^	771.5	MT, FE
Goethite	200	5.59	<1·10^-8^	<1·10^-8^	**5.86·10**^-4^	<1·10^-8^	<1·10^-8^	**0.6574**	<1·10^-8^	HM
Goethite	70	6.1	<1·10^-8^	<1·10^-8^	**5.86·10**^-4^	<1·10^-8^	<1·10^-8^	**1.273**	<1·10^-8^	HM
Goethite	22	6.22	<1·10^-8^	<1·10^-8^	**5.86·10**^-4^	<1·10^-8^	<1·10^-8^	**0.8475**	<1·10^-8^	HM
Magnetite	200	6.08	1.04·10^-5^	5.42·10^-6^	**5.82·10**^-4^	1.79·10^-6^	2.0·10^-5^	**0.653**	1.16·10^-3^	HM, MT
Magnetite	70	8.47	1.26·10^-4^	4.73·10^-5^	**5.04·10**^-4^	2.36·10^-8^	1.25·10^-5^	**1.094**	3.24·10^-5^	HM, MT
Magnetite	22	9.97	3.1·10^-4^	7.35·10^-5^	**3.98·10**^-4^	<1·10^-8^	4.26·10^-6^	**0.5759**	3.32·10^-6^	HM, MT
Ferrihydrite	200	5.59	<1·10^-8^	<1·10^-8^	**5.81·10**^-4^	<1·10^-8^	<1·10^-8^	**0.6512**	<1·10^-8^	HM
Ferrihydrite	70	6.1	<1·10^-8^	<1·10^-8^	**5.81·10**^-4^	<1·10^-8^	<1·10^-8^	**1.261**	<1·10^-8^	HM
Ferrihydrite	22	6.22	<1·10^-8^	<1·10^-8^	**5.81·10**^-4^	<1·10^-8^	<1·10^-8^	**0.8396**	<1·10^-8^	HM

Abbreviations: BN = bunsenite, NI = Ni metal, AW = awaruite, MT = magnetite, TT = tetrataenite, FE = Fe metal, HM = hematite.

We speculate that in the presence of Fe^0 ^most of the NH_4_^+ ^was rapidly formed in the first few hours of the experiment when unreacted surface was still available for reaction. In this scenario (e.g., run 2) most (and possibly all) H_2 _is formed *in situ *as a result of interactions between the pristine Fe^0 ^surface and H_2_O (Reaction 10) (e.g., *m*H_2 _= 0 at t_0_); however, the simultaneous Fe oxidation passivates the surface, reduces the availability of suitable H_2_/N_2 _sorption sites and the overall yield of the N_2_-reduction reaction.

(10) Fe + 2H_2_O → H_2 _+ Fe^2+ ^+ 2OH^-^

This notion is corroborated by experiments carried out with conditions in which H_2 _was present in the system from the start of the reaction (e.g., run 3), as a result of purging the solution with a H_2_/N_2 _mixture prior to loading (e.g., mH_2 _> 0 at t_0_). Abundant H_2 _in this run correlates with greater NH_4_^+ ^production as the overall N_2 _conversion rate increases from 2.5 to 10% (Tab. 2). Assuming that in both cases Fe^0 ^surface passivates at the same rate, then the H_2 _purged system produces more NH_4_^+ ^per unit of time because it does not depend on the Fe^0 ^alteration process (Reaction 10) to provide H_2_. This circumstance may be more typical of natural serpentinization-driven SHS where H_2 _can be provided by a number of processes, especially by Fe^2+ ^oxidation during alteration of rock-forming silicates [17,47].

Similar assumptions can be made about Ni_50_Fe_50 _and Ni_81_Fe_19 _assuming that Fe atoms exposed on the surface played a role in the reduction process. Due to good corrosion resistance, Ni^0 ^reacted to a much lesser extent and consistent with this lower activity is our experimental observation that the Ni^0 ^surface was not significantly altered (e.g., by precipitation of neoformed phases) throughout the experiment. Different modes of metal/alloy participation in studied reactions are discussed below.

The addition of KCl into the Fe^0^-H_2_O-N_2 _system in our experiments resulted in higher NH_4_^+ ^yield (Fig. 4a). While it may be intriguing to draw parallels with the Haber-Bosh process, where K is added to improve sticking coefficients and to help stabilize sorbed species [44], the apparent promoting effect of KCl may be partially or entirely caused by the presence of chloride ion (Cl^-^) in the solution. Cl^- ^can react with dissolved iron in the solution (Reaction 11) and remove products from the Fe^0 ^dissolution reaction (Reaction 10).

(11) Fe^2+ ^+ 2Cl^- ^→ FeCl_2_

Such a complexation reaction would result in an equilibrium shift towards the product side and further drive the dissolution process and release of structurally bound reduced N species into the solution (Reactions 3, 4). This is in agreement with the results of Reardon [48] who observed an increase in Fe^0 ^corrosion rates in low ionic strength (~0.02 *m*) anaerobic NaCl, NaHCO_3 _and Na_2_SO_4 _solutions compared to DI water. The NH_4_^+ ^content of the KCl reagent solution at concentrations used in our experiments was found to be below the detection limit of ion chromatography.

### Nitrite and Nitrate Reduction

Due to their status as environmental contaminants, NO_2_^-^/NO_3_^- ^reduction has been extensively studied, especially focused on the reduction of NO_3_^- ^by Fe^0^. Most of the published results concur that the reduction reaction (Reaction 12) at anaerobic ambient conditions exhibits the following set of features: 1) reaction rates decrease with increasing pH; 2) pH in unbuffered solutions becomes more alkaline as the reaction progresses; 3) NH_4_^+ ^and Fe_3_O_4 _are the dominant reaction products; 4) NO_3_^- ^reduction slows down once Fe^0 ^is coated with Fe_3_O_4 _unless Fe^2+^, Cu^2+^, Al^3+ ^or Fe^3+ ^are present; 5) SO_4_^2- ^inhibits the reaction; and 6) the molar N_2_:NH_4_^+ ^ratio in reaction products increases with pH [49-57].

(12) 4Fe^0 ^+ 10H^+ ^+ NO_3_^- ^→ NH_4_^+ ^+ 4Fe^2+ ^+ 3H_2_O

(13) 3Ni_3_Fe + 4NO_2_^- ^+ 24H_2_O → 32OH^- ^+ 4NH_4_^+ ^+ 3Fe^2+ ^+ 9Ni^2+^

Analogous reactions can be written for other metals/alloys as well as NO_2_^- ^(Reaction 13), although they have been by comparison less studied. The absence of resonance structures in the NO_2_^- ^molecule makes it easier to reduce than NO_3_^- ^[58], which is reflected in reduction reaction rates. For example, NO_3_^- ^reduction to NH_4_^+ ^in the presence of Fe^2+ ^was found to be a factor of 8 slower than that of NO_2_^- ^[5]. Because the reduction from NO_3_^- ^to NH_4_^+ ^requires transfer of at least 8 electrons, several intermediates must be formed in the process. Moreover, the formation of any N-N bonds must be avoided because it is effectively inert under all but the highest temperatures investigated here.

It is widely recognized, however, that NO_2_^- ^is a reaction intermediate in NO_3_^- ^reduction [e.g., [51]] [57,59]. For example, Wärna *et al *[59] proposed a reaction sequence from NO_3_^- ^and NO_2_^- ^through nitric oxide (NO), imidogen (HN˙) and aminyl radical (H_2_N¨) to NH_3_/NH_4_^+ ^on the surface of Fe^0^. Several studies with NO_2_^-^/NO_3_^- ^as well as some organic compounds suggest that Fe^2+^, Fe^0^, and Fe^2+ ^sorbed on neoformed Fe minerals (e.g., magnetite) are likely electron donors for the reduction reactions [e.g., [60]] [61,62]. The intriguing consequence of such a reaction mechanism in natural systems is that precipitation of secondary (neoformed) Fe minerals further along the flow path followed by surface sorption of Fe^2+ ^would provide additional reaction sites for the reduction of NO_2_^-^/NO_3_^- ^[e.g., [63]].

### The role of alloys/metals

Based on the XPS (oxidation state of Ni) and SEM (abundance of Fe- and the absence of Ni alteration phases) results it is possible to construct an order of apparent stability of studied alloys and metals

Ni^0 ^> Ni_81_Fe_19 _> Ni_50_Fe_50 _> Fe^0^

where Ni^0 ^is most- and Fe^0 ^is least stable under the studied experimental conditions. This enables us to generalize that the higher the Fe content, the higher the reactivity towards potential oxidizing agents (e.g., H_2_O, NO_2_^-^, NO_3_^-^) and thus the higher extent of alteration. Metals and alloys typically undergo reductive dissolution (e.g., Reaction 10); however alloys frequently dissolve the less noble metal preferentially, leaving the surface enriched in the more noble metal [e.g., [64]]. For example, the reductive dissolution of Ni_50_Fe_50 _alloys is expected to result in preferential release of Fe and a concomitant increase in the Ni:Fe ratio of the residual alloy.

Our findings are in agreement with metallurgical studies in which it has been demonstrated that Ni^0 ^is more corrosion resistant than Fe^0^, a notion that serves as a basis for their frequent alloying [38,65]. Unlike Fe^0^, Ni^0 ^reacts to a lesser degree in aqueous environments (reaction produces H_2 _and Ni^2+^), especially under reducing conditions. The presence of an oxidizing agent is usually required for significant corrosion; however, a protective oxide film may develop and impede further reactions [38,66]. Ni^0 ^with a combination of catalytic properties and corrosion resistance (e.g., slow dissolution kinetics) may serve as a basis for a unique mechanism of N_2 _reduction, where Ni acts both as a reactant and a catalyst. We hypothesize that Ni^0 ^reacts with H_2_O to produce H_2_, a portion of which may stay adsorbed on the surface in its atomic form (H_ads_) (Reaction 14). If N_2 _is also dissociatively chemisorbed (Reaction 15), surface-mediated reduction reactions may proceed (Reaction 16).

(14) Ni + 2H_2_O → 2H_ads _+ Ni^2+ ^+ 2OH^-^

(15) N_2_(aq) → 2N_ads_

(16) 2N_ads _+ 2H^+ ^+ 6H_ads _→ 2NH_4_^+^

This set of reactions may operate until all plausible sorption sites are exhausted and/or deactivated. By analogy, we argue that if the experimental conditions were approaching the stability field of Fe metal (e.g., at sufficiently high *f*H_2_) it could behave in a similar manner.

There exist; however, "true" catalytic systems for NO_2_^-^/NO_3_^- ^reduction, such as bimetallic Cu-Pt and Cu-Pd, Ag-Pd, Ag-Pt, which couple a noble metal and an oxidizable promoter. The reactions take place on the surface of Cu^0 ^which acts as an electron donor for the reduction of N species and as an acceptor of electrons from dissociative sorption of H_2 _on the surface of Pt [67,68]. Even though natural alloys of platinum group elements (Pt, Pd, Ir, Os, Rh, Ru) are scarce on modern Earth and are almost exclusively limited to magmatic segregation deposits, placers, and meteorites [e.g., [69]] [70,71], their significance for prebiotic synthesis should not be overlooked [72].

The predominantly alkaline pH in reacted samples (Tab. 2) is likely a result of several pH controlling reactions such as reductive dissolution of metals (e.g., Reaction 10), mineral formation (e.g., magnetite), and the buffering reactions involving charged species including, but not limited to NO_3_^-^, NO_2_^-^, NH_3_, or NH_4_^+^. The fate of Fe^2+ ^in the experiments reported here is difficult to constrain. Assuming completely anoxic conditions, temperatures below 85°C and a negligible *p*CO_2 _in our experiments, Fe(OH)_2 _(white rust) could precipitate (Reaction 17) and due to its instability serve as a precursor to other Fe oxides and hydroxides, most notably Fe_3_O_4 _(Reaction 18).

(17) Fe^++ ^+ 2OH^- ^→ Fe(OH)_2_

(18) 3Fe(OH)_2 _→ Fe_3_O_4 _+ 2H_2_O + H_2_

At higher temperatures and/or in the presence of trace levels of O_2 _or other oxidizing agents (e.g., NO_3_^-^), mineral intermediates such as green rust (mixed-valence hydroxide) may have been involved [e.g., [73]] [74,75].

The conversion of the original metal/alloy into a new mineral phase (e.g., coatings) may not necessarily negatively affect the NH_3_/NH_4_^+ ^production. For example, wüstite (FeO) and green rust – both possible reaction products/intermediates during anaerobic Fe^0 ^oxidation, have been shown to reduce NO_2_^-^/NO_3_^- ^[e.g., [50]] [76-78]. Magnetite and even goethite can also act as reductants for NO_2_^-^/NO_3_^-^, provided cations such as Fe^2+^, Cu^2+^, Fe^3+^, Al^3+ ^are present in the system [e.g., [53]] [56,79]. Equilibrium thermodynamic calculations, however, predict very little reactivity of magnetite, goethite, or ferrihydrite alone towards N_2 _and anaerobic experiments in the presence of green rust at ambient T, p conditions corroborate these predictions [80].

### Implications for the Hadean Earth

Different modes of metal/alloy participation have different implications for natural systems, especially in terms of the amount of metal/alloy required to achieve the same NH_3_/NH_4_^+ ^production. A catalyst remains stable during the reaction and therefore a small amount can, in theory, catalyze the formation of large amounts of NH_3_/NH_4_^+^. Conversely, a reactant would have to be present in sufficient amounts and/or would have to be continually formed in order to achieve comparable NH_3_/NH_4_^+ ^production. While both mechanisms are plausible on the Hadean Earth, it is hard to assess which of the two would be prevalent. Based of equilibrium geochemical modeling, Smirnov [28] concluded that at 200°C Ni metal is stable at *f*H_2 _orders of magnitude lower than Fe metal and even Ni_50_Fe_50 _(tetrataenite) and Ni_81_Fe_19 _(awaruite). Combined with results acquired from this study, it would appear that Ni metal is the most suitable candidate for a sustained long-term NH_3_/NH_4_^+ ^formation. Moreover, if we consider that the Hadean atmosphere may have had up to 30% H_2 _[29], the primordial ocean would contain significantly higher concentrations of dissolved H_2 _than today. In SHS, H_2 _from advected seawater combined with H_2 _formed by serpentinization could create conditions sufficiently reducing for stabilization of Fe containing alloys (e.g., awaruite, tetrataenite) and possibly even Fe^0^. Furthermore, as shown by Schoonen et al [72], seawater trapped in closed SHS (i.e., not open to seawater circulation) evolves to become extremely reduced as the partial pressure of hydrogen builds up.

**Figure 10:**
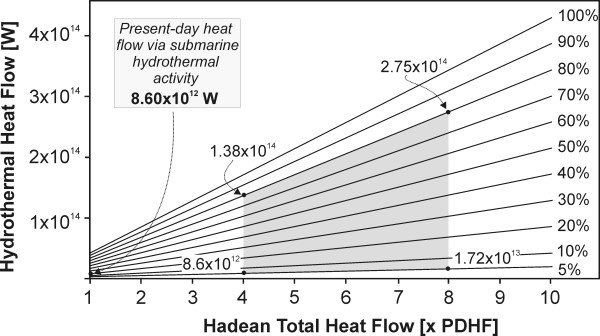
Hadean hydrothermal flow as a function of total Earth's heat flow (expressed as multiplicities of present day heat flow – PDHF). Each data line thus represents percentage of the total heat flow released through hydrothermal systems at a given value of Hadean heat flow. The shaded area represents assumed realistic scenarios for the Hadean.

The possibility of hydrothermal reduction of N_2 _to NH_4_^+ ^permits us to attempt to constrain the total NH_4_^+ ^flux from Hadean off-axis SHS. The following set of assumptions and variables were taken into account:

1) The total heat production of the Hadean Earth was several times higher than today [81,82]. Because the exact value is unknown, we calculated scenarios for 2- to 8-times the present day heat flow (PDHF = 4.3 × 10^13 ^W) [83] (Fig. 10); however, only values between 4 and 8 times PDHF are reported.

2) Because it is unclear if a global tectonic cycle was operational during the Hadean, we are unable to comment on the dissipation of Earth's internal heat, especially on the percentage of heat released through SHS. Therefore, two endmember scenarios are considered: a) heat is dissipated predominantly via volcanism (possibly through several supervolcanoes) and only 5% is released through hydrothermal activity; and b) 80% of heat is dissipated predominantly through hydrothermal activity (Fig. 10). For comparison, presently about 20% of PDHF is released through hydrothermal activity [83].

3) Due to the increased heat flow and possibly due to the blanketing effect of the atmosphere [e.g., [84]], we assume the mean ocean water temperature to be 70°C. Although modern serpentinization-driven SHS commonly vent fluids below 100°C [85-87], we assume that the higher overall heat flow in the Hadean would also increase the fluid temperature of hydrothermal vents [e.g., [88]]. The temperature of the discharging fluid is thus assumed to be 200°C for the purpose of this calculation. Although the temperature of ambient seawater and of discharging fluid directly influences the total hydrothermal fluid mass flux (equation 19), their variations (± 20°C) only produced small changes in the final NH_4_^+ ^fluxes (usually within the same order of magnitude; data not shown). The heat capacity (*c*_*p*_) of hydrothermal seawater at 200°C and P ~100–600 bar is 4.1 J.g^-1^K^-1^[89].

4) Ocean water is assumed to be in equilibrium with 1 bar of N_2_, resulting in a dissolved N_2_(aq) concentration of 0.481 mmol.kg^-1 ^at 70°C [90]. For simplicity, no other gases and/or aqueous ions were taken into consideration.

5) Even though experimental results reported in this study suggest a conversion of N_2_-to-NH_4_^+ ^0.2 to 2.5% we calculate a variety of scenarios ranging from 0.1% to 10%. Although the 10% conversion may appear overly optimistic, our experiments suggest that the presence of advected H_2 _and or K^+ ^may significantly improve the NH_4_^+ ^production (Tab. 2; Fig. 4a). Metals/alloys may act as either catalysts or reactants, however, if metals/alloys do react, it must be assumed that the rate of their destruction (e.g., passivation, poisoning) is equal to their rate of formation (e.g., via serpentinization). It is important to point out that for simplicity, we do not distinguish between respective metals/alloys and we are only concerned with their capability to facilitate the conversion of N_2 _to NH_4_^+ ^(in %).

The mass flux of seawater through hydrothermal systems (F) can be estimated from heat flux (H) in Watts, heat capacity of seawater (c_p _at 200°C) in J.g^-1^K^-1 ^and temperature anomaly ΔT in Kelvin [83]:

(19) $$F=\frac{H}{c_{p}\Delta T}.$$

**Figure 11:**
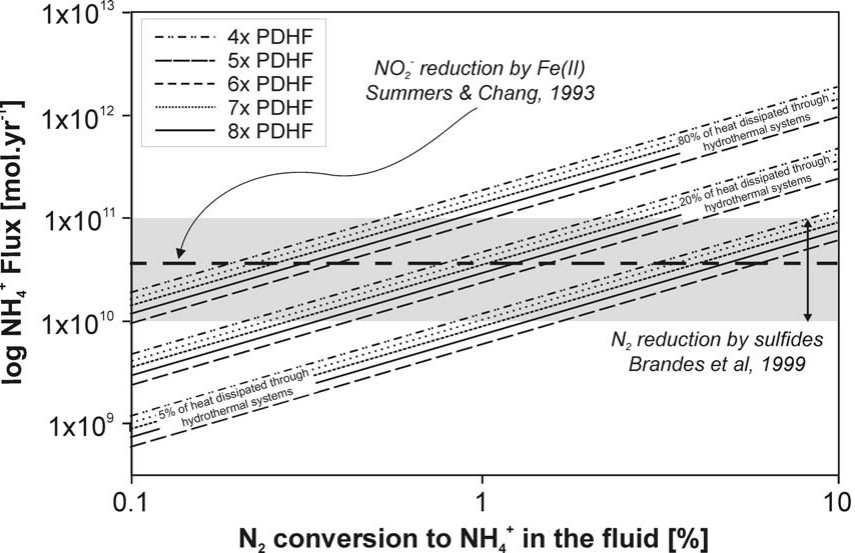
NH_4_^+ ^formation from N_2 _in Hadean hydrothermal systems. Fluxes are calculated as a function of N_2 _conversion between 1 and 10%. NH_4_^+ ^formation from NO_2_^-^/NO_3_^- ^is not included in these calculations.

Using hydrothermal heat fluxes from Fig. 10 we can calculate annual seawater mass fluxes from SHS. Subsequently, using various N_2_-to-NH_4_^+ ^conversion percentages (0.1 to 10%), annual NH_4_^+ ^production of Hadean SHS is calculated (Fig. 11). Assuming the most conservative scenario with 0.1% conversion of N_2 _to NH_4_^+^, the annual NH_4_^+ ^production would be between 5.9 × 10^8 ^mol (4 × PDTH) and 1.2 × 10^9 ^mol (8 × PDTH) if 5% of Earth's heat is removed via SHS and between 9.4 × 10^9 ^mol (4 × PDTH) and 1.9 × 10^10 ^mol (8 × PDTH) if 80% of heat is removed via SHS. Conversely, with a 10% N_2 _conversion efficiency, the annual NH_4_^+ ^production would be between 5.9 × 10^10 ^mol (4 × PDTH) and 1.2 × 10^11 ^mol (8 × PDTH) if 5% of heat is removed via SHS and between 9.4 × 10^11 ^mol (4 × PDTH) and 1.9 × 10^12 ^mol (8 × PDTH) if 80% of heat is removed by SHS (Tab. 5). To place these modeled fluxes in context we can compare their magnitude to those of other proposed NH_3_/NH_4_^+ ^formation mechanisms (Fig. 11). An annual NH_4_^+ ^flux at 1% conversion efficiency, for example, would be comparable to that based on a homogeneous reaction (Reaction 20) of Summers and Chang [4] or to the flux calculated by Brandes *et al *[13], which was based on NH_3 _formation in the presence of various minerals between 300 and 800°C.

**Table 5:** Total N content of four octahedrites analyzed by inert gas fusion (IMR Test Labs).

**Sample**	**N_TOTAL _[wt.%]**
Bogou Meteorite	0.0032 ± 0.0005
N'Goureyma Meteorite	0.0022 ± 0.0005
Sikhote Alin Meteorite	0.0032 ± 0.0005
Canyon Diablo Meteorite	0.0022 ± 0.0005

(20) 6Fe^2+ ^+ 7H^+ ^+ NO_2_^- ^→ 6Fe(III) + 2H_2_O + NH_3_

Although we cannot comment on the total NH_4_^+ ^content of the Hadean Ocean, we can estimate the contribution of hydrothermal N_2 _reduction per unit of time. For timescales longer than 1 year, the following equation may be used (21):

(21) $$NH_{4}^{+}\;addition\;to\;the\;ocean=\frac{annual\;NH_{4}^{+}\;production}{V_{Hadean\;Ocean}}•t.$$

**Figure 12:**
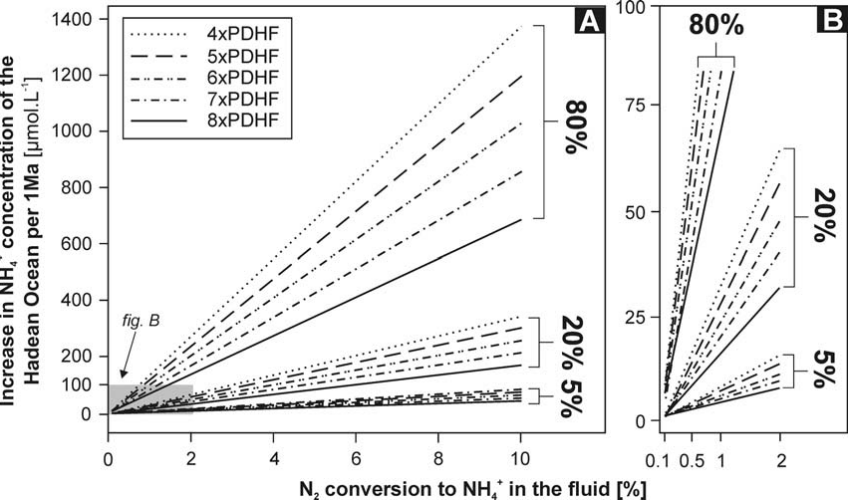
Estimated increase in NH_4_^+ ^concentration of the Hadean Ocean (in μmol.L^-1^) from the hydrothermal N_2 _reduction reaction per 1 Ma as a function of N_2_-to-NH_4_^+ ^conversion percentages, heat flow and percentage of heat released via hydrothermal systems (5, 20 and 80%).

NH_4_^+ ^production in mol.yr^-1 ^can be taken from Fig. 11 or Tab. 6 (or supplied from reader's own sources), *t *denotes the time period in years and V_ocean _is the total volume of Hadean Ocean in liters. We have calculated a scenario for one million years using the present-day global ocean volume (1.37 × 10^21 ^L) but alternative calculations can be quickly performed for different volumes (e.g., if the Hadean ocean had twice the volume of the present-day ocean, the NH_4_^+ ^concentrations in Fig. 12 and Tab 7 would be 50% smaller). For example, the conversion of 0.5% of N_2 _entrained in the advecting seawater would raise the NH_4_^+ ^content of the (completely homogenized) Hadean ocean by ~8 to 17 μmol.kg^-1 ^(4× to 8× PDHF) every 1 Ma (Tab. 7). These results (Fig. 12, Tab. 7) represent an upper contribution limit of this reaction, because no sinks (ion exchange, photooxidation, loss to the gas phase, formation of organic molecules, etc) were taken into account. In the absence of a comprehensive Hadean Nitrogen Cycle model, it is difficult to quantitatively assess the annual loss of NH_3_/NH_4_^+ ^from the ocean; however, the numbers in Fig. 12, Tab. 7 can be simply amended by assumption of loss expressed in %.

**Table 6:** NH_4_^+ ^formation from N_2 _in Hadean hydrothermal systems. Fluxes (mol.kg^-1^.yr^-1^) are calculated as a function of N_2 _conversion between 1 and 10%. Note that NH_4_^+ ^formation from NO_2_^-^/NO_3_^- ^is not included in these calculations.

5% of Earth's heat flow is released via hydrothermal activity					
*N*_2 _*conversion*	*Hadean heat flow (× PDHF)*				
%	4	5	6	7	8

0.1	5.9 × 10^8^	7.3 × 10^8^	8.8 × 10^8^	1.0 × 10^9^	1.2 × 10^9^
0.5	2.9 × 10^9^	3.7 × 10^9^	4.4 × 10^9^	5.1 × 10^9^	5.9 × 10^9^
1	5.94 × 10^9^	7.3 × 10^9^	8.8 × 10^9^	1.0 × 10^10^	1.2 × 10^10^
2	1.2 × 10^10^	1.5 × 10^10^	1.8 × 10^10^	2.1 × 10^10^	2.4 × 10^10^
5	2.9 × 10^10^	3.7 × 10^10^	4.4 × 10^10^	5.1 × 10^10^	5.9 × 10^10^
10	5.9 × 10^10^	7.3 × 10^10^	8.8 × 10^10^	1.0 × 10^11^	1.2 × 10^11^

20% of Earth's heat flow is released via hydrothermal activity					

*N*_2 _*conversion*	*Hadean heat flow (× PDHF)*				
%	4	5	6	7	8

0.1	9.4 × 10^9^	1.2 × 10^10^	1.4 × 10^10^	1.7 × 10^10^	1.9 × 10^10^
0.5	4.7 × 10^10^	5.9 × 10^10^	7.1 × 10^10^	8.2 × 10^10^	9.4 × 10^10^
1	9.5 × 10^10^	1.2 × 10^11^	1.4 × 10^11^	1.7 × 10^11^	1.9 × 10^11^
2	1.9 × 10^11^	2.4 × 10^11^	2.8 × 10^11^	3.3 × 10^11^	3.8 × 10^11^
5	4.7 × 10^11^	5.9 × 10^11^	7.1 × 10^11^	8.2 × 10^11^	9.4 × 10^11^
10	9.4 × 10^11^	1.2 × 10^12^	1.4 × 10^12^	1.7 × 10^12^	1.9 × 10^12^

80% of Earth's heat flow is released via hydrothermal activity					

*N*_2 _*conversion*	*Hadean heat flow (× PDHF)*				
%	4	5	6	7	8

0.1	9.4 × 10^9^	1.2 × 10^10^	1.4 × 10^10^	1.7 × 10^10^	1.9 × 10^10^
0.5	4.7 × 10^10^	5.9 × 10^10^	7.1 × 10^10^	8.2 × 10^10^	9.4 × 10^10^
1	9.5 × 10^10^	1.2 × 10^11^	1.4 × 10^11^	1.7 × 10^11^	1.9 × 10^11^
2	1.9 × 10^11^	2.4 × 10^11^	2.8 × 10^11^	3.3 × 10^11^	3.8 × 10^11^
5	4.7 × 10^11^	5.9 × 10^11^	7.1 × 10^11^	8.2 × 10^11^	9.4 × 10^11^
10	9.4 × 10^11^	1.2 × 10^12^	1.4 × 10^12^	1.7 × 10^12^	1.9 × 10^12^

**Table 7:** Estimated increase in NH_4_^+ ^concentration of the Hadean Ocean (in μmol.L^-1^) from the hydrothermal N_2 _reduction reaction per 1 Ma as a function of N_2_-to-NH_4_^+ ^conversion percentages, heat flow and percentage of heat released via hydrothermal systems.

5% of Earth's heat flow is released via hydrothermal activity					
*N*_2 _*conversion*	*Hadean heat flow (× PDHF)*				
%	4	5	6	7	8

0.1	0.4	0.5	0.6	0.8	0.9
0.5	2.1	2.7	3.2	3.8	4.3
1	4.3	5.4	6.4	7.5	8.6
2	8.6	10.7	12.9	15.0	17.2
5	21.4	26.8	32.2	37.5	42.9
10	42.9	53.6	64.3	75.0	85.8

20% of Earth's heat flow is released via hydrothermal activity					

*N*_2 _*conversion*	*Hadean heat flow (× PDHF)*				
%	4	5	6	7	8

0.1	1.7	2.1	2.6	3.0	3.4
0.5	8.6	10.7	12.9	15.0	17.2
1	17.2	21.4	25.7	30.0	34.3
2	34.3	42.9	51.5	60.0	68.6
5	85.8	107	129	150	172
10	172	214	257	300	343

80% of Earth's heat flow is released via hydrothermal activity					

*N*_2 _*conversion*	*Hadean heat flow (× PDHF)*				
%	4	5	6	7	8

0.1	6.9	8.6	10.3	12.0	13.7
0.5	34.3	42.9	51.5	60.0	68.6
1	68.6	85.8	103	120	137
2	137	172	206	240	274
5	343	429	515	600	686
10	686	858	1030	1200	1370

It is imperative to note that due to a large number of unknown and/or poorly constrained variables, these calculations should only be regarded as a first order approximation. However, it is clear that N_2 _reduction, albeit very inefficient, could have been a significant source of NH_4_^+^, especially in localized environments.

Because NO_2_^-^/NO_3_^- ^are inherently easier to reduce than N_2_, its presence in advected seawater could have significantly increased the annual NH_4_^+ ^hydrothermal flux. It is unclear; however, how much NO_2_^-^/NO_3_^- ^would be advected into the crust, especially in the presence of such significant sinks as the reduction by Fe(II) [5]. While this process is a viable pathway to abiotic NH_3_/NH_4_^+^, its operation is dependent on atmospherically-driven processes of NO_2_^-^/NO_3_^- ^formation as well as chemical composition of the Hadean Ocean, especially pH and *m*Fe^2+^. A change in one of the parameters (e.g., a shift in oceanic pH) may have negatively affected or completely halted this pathway. We assume that serpentinization-driven SHS would have been less affected by changes in ocean water chemistry because their physico-chemical conditions (e.g., pH, *f*H_2_) are determined by fluid-rock interactions (e.g., availability of fresh rock) and possibly magmatic input rather than ocean composition. Moreover, the high temperature and pressure conditions combined with accumulations of suitable minerals would make these environments well suited for a long term, sustained NH_3_/NH_4_^+ ^production on the Hadean Earth.

Besides facilitating the production of NH_3_/NH_4_^+^, metals and alloys in SHS may have been involved in other reactions potentially important for prebiotic synthesis. In the context of environmental science, for example, Fe^0 ^was found to reduce nitrobenzene [91] or to facilitate reductive dehalogenation of carbon tetrachloride and chloroform [92-94]; Fe^2+ ^sorbed on Fe(III) minerals decomposes nitrobenzene [60]. Our future research will also assess SHS as potential sinks of prebiotic molecules during the late Hadean/early Archaean.

The notion that N is commonly present in Fe^0 ^in its reduced form presents a possibility of meteoritic delivery of reduced N species to Earth, especially during the periods of heavy bombardment. Fe^0 ^and its alloys (e.g., tetrataenite, awaruite, kamacite) are among the dominant mineral phases in iron meteorites and to a lesser extent in stony-iron meteorites [e.g., [95]] [96-99].

To assess the possible importance of meteoric delivery of reduced N to Earth, we submitted four octahedrites for inert gas fusion analyses (IMR Test Labs, Lansing, NY). The meteorites – Bogou (IAB), Sikhote Alin (IIAB), Canyon Diablo (IAB), N'Goureyma (Ungrouped) [100-103] (Stony Brook University's meteorite collection) contained 22 to 32 ppm of N_TOT _(Tab. 5). Although these analyses provided no insight into the oxidation state or speciation of nitrogen in these meteorites, it is likely to be present predominantly in the form of nitride (N^3-^) as is the case in similar commercially available metals, alloys and known meteorite minerals (e.g., roaldite, carlsbergite). Hence in the following calculation we assume that all meteorite-associated nitrogen is present as nitride. Nitride would be readily released from meteorites after falling into the Hadean Ocean as a result of the rapid and complete dissolution due to inherent instability of Fe^0 ^in aqueous solutions (even in O_2_-free solutions). Aqueous nitride is expected to react quickly with protons to form NH_3_/NH_4_^+^. Similar scenario for meteoritic delivery of phosphorus has already been proposed by Pasek and collaborators [104,105].

To constrain the influx of meteoritic N to the Hadean Ocean we have adapted the meteorite flux values used by Pasek *et al *[104,105]: 2 × 10^5 ^kg.year^-1 ^(current flux of iron meteorites to Earth; 50% of total meteoritic flux by weight) and meteoritic flux 10^5^-10^6 ^times the present-day value during the Late Heavy Bombardment Period. Using these values we have created models for varying total reduced N content of iron meteorites: 5, 10, 15, 20, and 30 ppm. For comparison, average and median values for N_TOT _from 91 published analyses [106,107] and four analyses acquired in this study were 20.1 and 12.3 ppm respectively.

**Figure 13:**
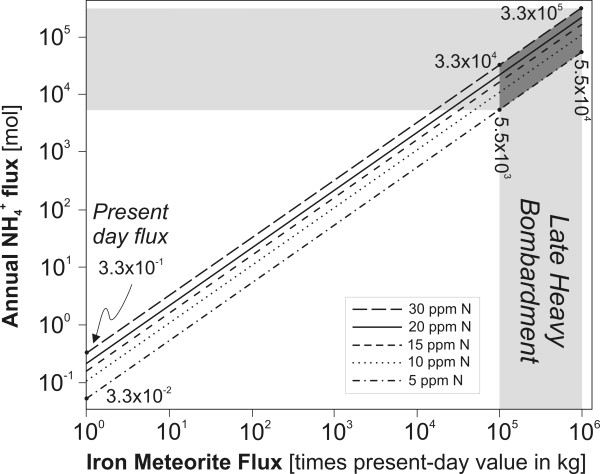
Estimated annual flux of nitrogen from iron meteorites. Different line styles correspond to different average N content of iron meteorites. Shaded areas represent iron meteorite flux during the period of Late Heavy Bombardment.

The results presented in Fig. 13 show that during the Late Heavy Bombardment Period, iron meteorites could have delivered ~10^3 ^to 10^5 ^mol.yr^-1 ^of N_TOT _to Earth which is approximately six to nine orders of magnitude less than our estimates for hydrothermal production (Fig. 11). Although the N influx was likely negligible on the global scale it is possible that crater lakes associated with iron meteorite impacts [108] may have contained significant concentrations of NH_3_/NH_4_^+^. Environments containing NH_3_/NH_4_^+ ^and organic phosphorous compounds (e.g., phosphonates, organophosphates) from corroding iron meteorites ((Fe,Ni_3_)P) [104,105] thus could have created very favorable, spatially-restricted conditions for prebiotic synthesis, perhaps unparalleled on the prebiotic Earth.

## Conclusion

1) N_2 _reduction to NH_4_^+ ^was found to be limited (up to 2.5% at 200°C) compared to NO_2_^-^/NO_3_^- ^(100% at 200°C)

2) Metals are more effective at reducing NO_2_^-^/NO_3_^- ^than alloys; NH_4_^+ ^is the dominant reaction product.

3) The reduction process exhibits a strong temperature dependence.

4) Fe^0 ^and Ni^0 ^were found to be least- and most resistant to alteration, respectively.

5) Ni^0^, Fe^0^, Ni_50_Fe_50_, Ni_81_Fe_19 _were found to contain up to 124 ppm of nitrogen in their structures, some of which is released upon dissolution and reacts to form NH_4_^+^.

6) Serpentinization-driven SHS were likely important sources of abiotic NH_3_/NH_4_^+ ^in the Hadean Ocean.

## Competing interests

The authors declare that they have no competing interests.
